# Survival and disease burden of breast cancer (Monastir, Tunisia): A population-based cohort study (2002–2022)

**DOI:** 10.1371/journal.pone.0360398

**Published:** 2026-10-14

**Authors:** Imen Zemni, Amani Maatouk, Wiem Boufrikha, Cyrine Bennasrallah, Wafa Dhouib, Meriem Kacem, Hela Abroug, Manel Ben Fredj, Inès Bouanene, Islem Jridi, Hiba Arfa, Amina Ben Salem, Leila Njim, Sonia Zaied, Raja Faleh, Karawen Sakka, Badreddine Sriha, Lilia Jaîdane, Meriem Mehdi, Asma Belguith Sriha

**Affiliations:** 1 Department of Epidemiology and Preventive Medicine, Fattouma Bourguiba Hospital of Monastir, University of Monastir, Faculty of Medicine of Monastir, Monastir, Tunisia; 2 Technology and Medical Imaging Research Laboratory - LTIM - LR12ES06, University of Monastir, Faculty of Medicine of Monastir, Monastir, Tunisia; 3 Department of Hematology, University maternity Center of Monastir, University of Monastir, Faculty of Medicine of Monastir, Monastir, Tunisia; 4 Department of Hospital Hygiene, Fattouma Bourguiba Hospital of Monastir, University of Monastir, Faculty of Medicine of Monastir, Monastir, Tunisia; 5 Department of community and Preventive Medicine Regional Hospital of Ksar Hellal, University of Monastir, Faculty of Medicine of Monastir, Monastir Tunisia; 6 Department of Family Medicine, University of Monastir, Faculty of Medicine of Monastir, Monastir, Tunisia; 7 Department of Radiology, University Maternity Center of Monastir, University of Monastir, Faculty oF Medicine of Monastir, Monastir, Tunisia; 8 Department of Pathology, Fattouma Bourguiba Hospital of Monastir, University of Monastir, Faculty of Medicine of Monastir, Monastir, Tunisia; 9 Department of Oncology, University Maternity Center of Monastir, University of Monastir, Faculty of Medicine of Monastir, Monastir, Tunisia; 10 Department of Gynecology, University Maternity Center of Monastir, University of Monastir, Faculty of Medicine of Monastir, Monastir, Tunisia; 11 Cancer Registry of the Center of Tunisia, University Hospital Farhat Hached of Sousse, University of Sousse, Sousse, Tunisia; 12 Department of Reproductive Biology, University Maternity Center of Monastir, University of Monastir, Faculty of Medicine of Monastir, Monastir, Tunisia; Faculty of Medicine of Tunis, TUNISIA

## Abstract

**Background:**

Population-based evidence on Breast Cancer (BC) survival and disease burden in Tunisia remains limited. This study aimed to describe BC survival and temporal trends, quantify the BC burden, and assess the impact of early diagnosis on survival and burden.

**Methods:**

We conducted a descriptive population-based cohort study of women diagnosed with BC in Monastir between 2002 and 2014, with follow-up through January 2022. Overall survival was estimated using Kaplan-Meier methods, and factors associated with mortality were assessed using multivariable Cox regression. BC burden was quantified using disability-adjusted life years (DALYs), comprising years of life lost (YLLs) and years lived with disability (YLDs). Relative risks (RRs) and related measures were used to assess the association between stage at diagnosis and mortality.

**Results:**

Among 1,075 incident BC cases identified between 2002 and 2014, 21.4% were diagnosed at stage IV. By 2022, vital status information was available for 959 women. The crude and age-standardized mortality rates were 11.9 (95% CI, 8.5–15.4) and 12.5 (95% CI, 8.9–16.0) per 100,000 WY, respectively. Median survival was 187 months (95% CI, 163.7–210.2), reaching 226 months among women diagnosed at stages 0–I. Three-, five-, and ten-year survival rates were 77.0%, 70.0%, and 45.2%, respectively. Ten-year relative survival showed a significant upward trend among women aged 30–64 years, corresponding to the target population of the national BC early detection program (APC, 5.37%; 95% CI, 2.03–8.82; p = 0.005). The total BC burden was 10,665.4 DALYs, including 10,362 YLLs (97.3%). Median DALYs per patient was 28.0 years (IQR, 13.74–36.95). Diagnosis at stages 0–II was associated with a 22% reduction in ten-year mortality (RR, 0.78; 95% CI, 0.67–0.91), with an NND of 9. Diagnosing 61 women at stages 0–II rather than III–IV would avoid one DALY.

**Conclusion:**

The significant upward trend in ten-year survival among women targeted by the national BC early detection program, together with the avoidance of one DALY per 61 women diagnosed at stages 0–II rather than III–IV, supports continued investment in early detection and BC control in Tunisia.

## Introduction

### Background/rationale

Breast Cancer (BC) is the most frequently diagnosed cancer and one of the leading causes of cancer-related mortality among women worldwide [1]. In Tunisia, as in many low- and middle-income countries, BC incidence has continued to increase [2,3], placing a growing burden on the healthcare system [4]. During the study period, a national BC control program was implemented in Tunisia, with a primary focus on early detection through clinical breast examination (CBE) among women aged 30–64 years. This approach reflected the limited availability of mammography facilities, which constrained the feasibility of implementing a nationwide mammography-based screening program comparable to those established in high-income countries [5]. Early detection was therefore promoted through CBE, with mammographic assessment used as an adjunct for women with clinical or individual risk factors. In particular, mammography was recommended for women with a maternal family history of BC and for women aged over 45 years who had access to and could afford mammographic screening.

Furthermore, to address the growing BC cancer burden, Tunisia implemented three population-based cancer registries that aimed at strengthening cancer surveillance, monitoring, and evaluation systems. Cancer survival is considered as a major indicator for evaluating the effectiveness of cancer control strategies, including early detection, therapeutic management, and care pathways [2,3]. These registries have enabled the assessment of cancer incidence and trends in Tunisia; however, population-based studies evaluating BC survival remain limited, and data on disease burden are scarce. Assessing both survival and BC burden is important, given that data in Tunisia remain limited [6,7]. Therefore, a population-based study is needed to provide updated estimates of BC survival, assess temporal trends, quantify the disease burden, and evaluate the potential impact of early diagnosis strategies on survival and burden in the Tunisian population.

**Objectives**: This study aimed to describe BC survival rates and temporal trends, quantify the disease burden in Monastir from 2002 to 2022, and assess the impact of early diagnosis on survival and disease burden.

## Methods

### 1. Study design

We conducted a descriptive population-based cohort, tracking all BC patients diagnosed between 2002 and 2014 in the governorate of Monastir (Tunisia), with follow-up until January 2022. Our study protocol has been deposited in protocols.io [8].

### 2. Setting

The governorate of Monastir, located in central-eastern Tunisia, had approximately 287,615 female inhabitants in 2014, including 118,822 women aged 30–64 years. During the study period, healthcare access was characterized by a physician density of approximately one physician per 1,666 inhabitants and one tertiary university hospital, Fattouma Bourguiba University Hospital, comprising two main units (A and B). The B unit had a dedicated oncology department established in 2012 and radiology department with mammography services [9]. Gynecologists from both the public and private sectors, together with volunteer primary care physicians, have played an active role in the early detection of BC program since its inception. Approximately 30–40 midwives were actively involved in the national BC control program during the study period, primarily through CBE. All cancer cases occurring among Monastir residents, regardless of the location of diagnosis or treatment, were recorded in the Cancer Registry of the Central Region [10]. Patients with confirmed BC were managed by multidisciplinary teams including gynecologists, oncologists, and radiotherapists, according to established treatment protocols.

### 3. Participants

Inclusion criteria: This study encompassed all cases of BC diagnosed in patients residing in Monastir between January 2002 and December 2014. Participant follow-up was ensured through linkage with the Monastir all-cause mortality database, spanning the years 2002 to January 2022.

Non-inclusion criteria: Benign and in situ tumors, tumor recurrences, nor any cases of disease relapse or metastatic progression.

### 4. Variables

Malignant neoplasms of the breast were classified according to the 10^th^ edition of the International Classification of Diseases (ICD-10), with code C50 (C50.0–C50.9). Data included demographic characteristics (age, sex), baseline clinic-pathological variables (localizations, histological types, stage) date of BC diagnosis, death status and date of death.

### 5. Data sources/measurements

The data source of BC cases in both the public and private sectors was the Cancer Registry of the Centre, from which data relating to Monastir region was selected [2]. BC cases from both public and private sectors were obtained from the Central Cancer Registry, with data for the Monastir region extracted for this study [2]. Access to the merged database was obtained in late 2019. Vital status (alive or deceased) was verified using multiple sources, including hospital discharge records and the official death registry from the Ministry of Social Affairs database (access granted in February 2023), with verification at the municipal level to ensure completeness [10]. Patients whose vital status could not be definitively ascertained by the end of follow-up were considered lost to follow-up.

Histological subtypes were classified according to ICD-O-3 morphology codes and grouped into invasive ductal carcinoma (8500, 8501, 8503, 8523), invasive lobular carcinoma (8520, 8522), mucinous carcinoma (8480), Paget disease of the nipple with invasive carcinoma (8541). Tumor topography was classified according to the ICD-O-3 topography codes. Peripheral localizations, including the nipple (C50.0), central portion (C50.1), upper-inner quadrant (C50.2), lower-inner quadrant (C50.3), upper-outer quadrant (C50.4), lower-outer quadrant (C50.5), and axillary tail (C50.6); overlapping (multifocal) localizations (C50.8); and unspecified breast localizations (C50.9). Tumor stage was classified according to the TNM staging system of the *AJCC Cancer Staging Manual*, 8th edition [11]. Stage was missing for 74 patients (6.9%). To assess potential selection bias, Table S1 in S1 Appendix compares patients with missing and complete stage data according to age, tumor localization, histological subtype, and year of diagnosis. No significant differences were observed between the two groups (all p > 0.05).

To mitigate the potential impact of unavailable vital status information on the crude mortality rate (CMR), a sensitivity analysis was performed. To obtain an adjusted, age-specific mortality rate, the patients with unknown vital status were stratified by age group in Excel. Within each age group, the observed probability of death in patients with known vital status was applied to those with unknown vital status to estimate the number of expected deaths. Estimated deaths = number of patients with missing vital status × observed mortality proportion. The estimated deaths were then added to the observed deaths. Precision was assessed using 95% confidence intervals (95% CI), calculated as 95% CI = $p\pm \text{1}.\text{9}\text{6}\times \sqrt{\frac{p\times (\text{1}-p)}{n}}$, where p represents the observed mortality rate and n the sample size [12]. Age-Standardized Mortality Rates (ASMR) were computed as weighted averages of age-specific mortality rates using the corresponding weight in the standard population (World Standard Population) [13] (Table S2 in S2 Appendix).

The median survival was defined as the time from diagnosis or treatment initiation at which 50% of patients remained alive [14]. The three-, five- and ten-year survival rates represent the probability of patients remaining alive 3, 5 or 10 years, respectively, following BC diagnosis or treatment [15]. These rates, expressed as percentages, reflect the proportion of patients surviving.

Disability-adjusted life years (DALYs) were estimated as the sum of years of life lost (YLLs) and years lived with disability (YLDs). YLLs were calculated by multiplying the number of deaths by the remaining life expectancy at the age of death using the Tunisian national life expectancy at birth (79 years for females and 74.2 years for males) [16], YLDs were estimated using an incidence-based approach. For each patient, YLD was calculated as the product of the observed follow-up duration (from diagnosis to death or last follow-up) and a stage-specific disability weight assigned according to the clinical stage at diagnosis. (${YLD}_{i}=\;{DW}_{i}\times \;D_{i}\;$ and $YLD=\sum_{i=\text{1}}^{n}{YLD}_{i}$). Disability weights were obtained from the multicenter study by Yan et al. and were 0.162, 0.158, 0.176, and 0.265 for stages I, II, III, and IV, respectively [17]. Total DALYs were obtained by summing YLLs and YLDs across all patients [18–20]. Relative risks (RRs), relative risk reductions (RRRs), and numbers needed to diagnosis (NND) were calculated to quantify the association between stage at diagnosis and mortality. RRs were estimated from the risks of death in the early-stage (0–II) and advanced-stage (III–IV) groups; RRR was calculated as 1 − RR, and NND as 1/absolute risk reduction (ARR).

### 6. Statistical methods

Data were entered using Microsoft Excel and analyzed with IBM SPSS Statistics version 21.0 (IBM Corp., Armonk, NY, USA). Continuous variables were summarized as medians and interquartile ranges (IQRs), whereas categorical variables were expressed as frequencies and percentages. Baseline characteristics of patients with complete and missing follow-up were compared using the Chi-square test. Cancer-specific survival was estimated using the Kaplan–Meier method and compared using the log-rank test. Median survival times and three-, five-, and ten-year survival rates were reported with the corresponding 95% confidence intervals (95% CIs). The standard errors of the Kaplan–Meier survival estimates were calculated using Greenwood’s formula. A multivariable Cox proportional hazards analysis was performed, including baseline clinic-pathological variables considered potentially associated with mortality: age groups (< 40, 40–64 and >64 years), stage (0-I; II, III and IV), histological types (invasive ductal carcinoma, invasive lobular carcinoma, mucinous carcinoma, paget disease of the nipple), localizations (nipple &central localizations, overlapping localizations, outer quadrant, inner quadrant). The proportional hazards assumption was assessed graphically using log-minus-log survival plots. Crude mortality rates (CMRs) and age-standardized mortality rates (ASMRs) were calculated per 100,000 person-years with 95% CIs. Three-five-and ten-year survival were estimated using the Life Tables/Survival Analysis procedure in SPSS and the standard errors were used to derive the corresponding 95% confidence intervals. Temporal trends in survival rates, were evaluated using Joinpoint Regression Program (version 5.4.0), and annual percent changes (APCs) with corresponding 95% CIs were estimated [21]. The burden of disease was assessed by calculating years of life lost (YLLs), years lived with disability (YLDs), and disability-adjusted life years (DALYs) for each patient. Continuous burden indicators (YLLs, YLDs, and DALYs) were summarized as medians and IQRs because of their skewed distributions.. Statistical significance was set at a two-sided p value <0.05.

### Ethics approval and consent to participate

The study was conducted according to ethical standards collections. To maintain the principle of confidentiality, the data used were anonymized [8] The study approval was obtained from the Ethics Committee of the Faculty of Medicine of Monastir (approval number IORG 0009738 N°101/ OMB 0990−0279).

## Results

### A. Breast cancer in females

Between 2002 and 2014, a total of 1,075 incident BC cases, were identified. Of these, 959 women (89.2%) were included in the survival analysis. The median follow-up time was 12 years (IQR:9–15). Comparison of patients with complete and missing follow-up data revealed no significant differences in age, disease stage, histological subtype, or tumor localization. Patients diagnosed during 2002–2004 had 12.7% of missing vital status data compared with those diagnosed during 2011–2014 (3.6%) (p = 0.001) (Table 1).

**Table 1:** Comparison of patients with missing and complete follow-up data (2002-2022 Monastir, Tunisia).

	Missing data	Valid data	p
**All**	116 (10.8)	9591 (89.2)	
**Age groups**			
< 30	2 (5.6)	34 (94.4)	0.168
30-39	13 (6.6)	184 (93.4)	
40-49	10 (3.2)	300 (96.8)	
50-59	15 (6.1)	231 (93.9)	
69	10 (6.8)	136 (93.2)	
≥ 70	2 (1.9)	106 (98.1)	
**BC stage**			
0-1	5 (3.8)	128 (96.2)	0.529
2	21 (4.3)	466 (95.7)	
3	3 (1.8)	164 (98.2)	
4	8 (3.7)	206 (96.3)	
**BC histological tumor types**			
Invasive ductal carcinoma (IDC)	44 (6.3)	658 (93.7)	0.994
Others BC histological tumor types	8(6.1)	123 (93.9)	
**BC localizations**			
Nipple &central localizations	2 (5.6)	34 (94.4)	0.267
Upper-inner quadrant	6 (6.6)	85 (93.4)	
Lower-inner quadrant	4 (10.0)	36 (90.0)	
Upper-outer quadrant	18 (7.1)	235 (92.9)	
Lower-outer quadrant	5 (8.3)	55 (91.7)	
Overlapping localizations	8 (3.1)	252 (96.9)	
**Year of diagnosis**			
2002-2004	20 (12.7)	137 (87.3)	**0.001**
2005-2007	18 (7.7)	216 (92.3)	
2008-2010	15 (5.2)	276 (94.8)	
2011-2014	14 (3.6)	395 (95.2)	

For BC histological tumor types and BC localizations 242 of data were missing (22.5%).

BC was diagnosed at stage IV in 21.4% of all cases, including 18.8% of patients aged 30–64 years and 33.3% of those aged > 64 years (p < 0.0001) (Fig 1).

**Fig 1:**
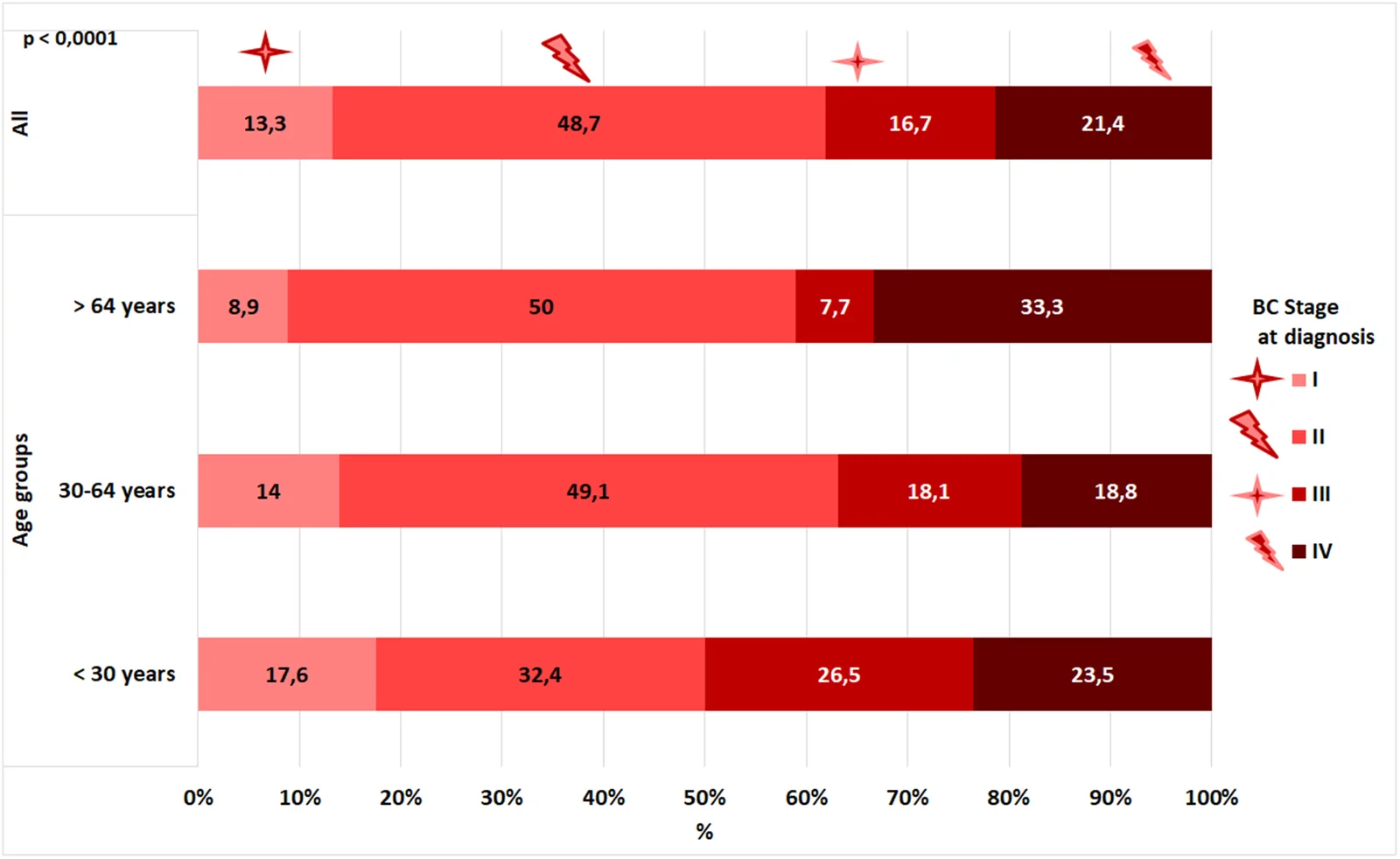
Distribution of breast cancer stages at diagnosis according to age group. The proportion of women diagnosed with stages 0–II BC was 62% overall. Among women aged >64 years, 33.3% were diagnosed with stage IV disease. The distribution of BC stage differed significantly across age groups (p < 0.0001).

The median age at diagnosis was 49 years (IQR: 41–59). The average of CMR and ASMR per 100,000 WY, were 11.9 (95% CI: 8.5–15.4) and 12.5 (95% CI: 8.9–16.0), respectively. Histologically, invasive ductal carcinoma was the predominant subtype (84.2%), followed by invasive lobular carcinoma (6.1%), mucinous carcinoma (1.7%), and Paget disease of the nipple with invasive carcinoma (2.0%), while other histological subtypes collectively accounted for 5.4% of cases (S3 Appendix). Regarding tumor topography, the outer quadrant was the most common BC localization, accounting for 41.6% of cases (Table 2).

**Table 2:** Mortality rates and median survival by age and pretreatment breast cancer characteristics (2002-2022 Monastir, Tunisia).

		Mortality rates		Median survival	
	**N**	**N (%)**	**CMR**^**µ**^ **(95% CI)**	**N’**	**Median (months) (95% CI)**
**All Females**	959	446/959 (46.5)	11.9 (8.5-15.4)	947	187 (163.7-210.2)
***ASMR***			**12.5 (8.9-16.0)**		
**Age groups**	945	444			
< 30	34 (3.6)	8 (23.5)	0.42 (0-1.06)	34	–
30-40	179 (18.9)	67 (37.4)	11.02 (7.7-14.34)	177	–
40-49	288 (30.2)	102 (34.0)	23.0 (18.2-27.7)	287	–
50 −59	215 (22.8)	91 (39.4)	24.7 (19.7-29.7)	214	210 (153.9-266.1)
60-69	127 (13.4)	78 (57.4)	35.8 (29.8-41.8)	126	119 (96.2-141.7)
≥ 70	102 (10.8)	98 (92.5)*	53.7 (46.4-61.1)	101	49 (33.8-64.2)*
**BC stage**	928	440			
0-1	120 (12.9)	49 (38.3)		120	226.0 (113.8-338.2)
2	454 (48.9)	200 (42.9)		450	202.0†
3	156 (16.4)	59 (36.0)		155	207.0 (170.5-243.5)
4	198 (21.3)	132 (64.1)		196	78.0 (54.8-101.2)*
**BC histological tumor types**	736	372			
Invasive ductal carcinoma (IDC)	621 (84.2)	322 (51.5)		617	143 (114.7-171.3)
Invasive lobular carcinoma (ILC)	45 (6.1)	19 (42.2)		44	–
Mucinous carcinoma	14 (1.7)	6 (42.9)		13	150.0
Paget disease of the nipple with invasive carcinoma	16 (2.0)	6 (37.5)		16	
Others histological types	40 (5.4)	19 (45.2)		40	190 (112.9-267.1)
**BC localizations**	663	330			
Nipple &central localizations	33 (5.0)	22 (66.7)		32	49 (23.5-74.5)
Upper-inner quadrant	80 (12.1)	39 (48.8)		80	142 (120-160)
Lower-inner quadrant	35 (5.3)	18 (51.4)		35	199 (76.0-322.0)
Upper-outer quadrant	226 (34.1)	98 (43.2)		223	201.0 (140.2-374.8)
Lower-outer quadrant	50 (7.5)	23 (46.0)		50	126.0 (90.8-161.2)
Overlapping localizations	239 (36.0)	130 (53.9)		237	156.0 (130.6-181.4)*

N: Population included; 95% CI: 95% confidence interval, BC: Breast Cancer; -:Survival median not reached; † Confidence interval not calculated; *: p < 0.05; µ: Mortality rates per 100,000 WY; CMR: crude mortality rates, ASMR: age standardized mortality rates. N’: N:population at risk in survival analysis.

#### 1. Breast cancer survival analysis.

The median survival was 187 months (95% CI: 163.7–210.2). It was was 210 months among women aged 50–59 years. Median survival was 226 months among women with BC stage 0–I and 201 months among those with tumors located in the upper-outer quadrant (Table 2).

According to the Kaplan–Meier method, BC median survival decreased with increasing age, with increasing BC stage, and according to tumor localization (Fig 2).

**Fig 2:**
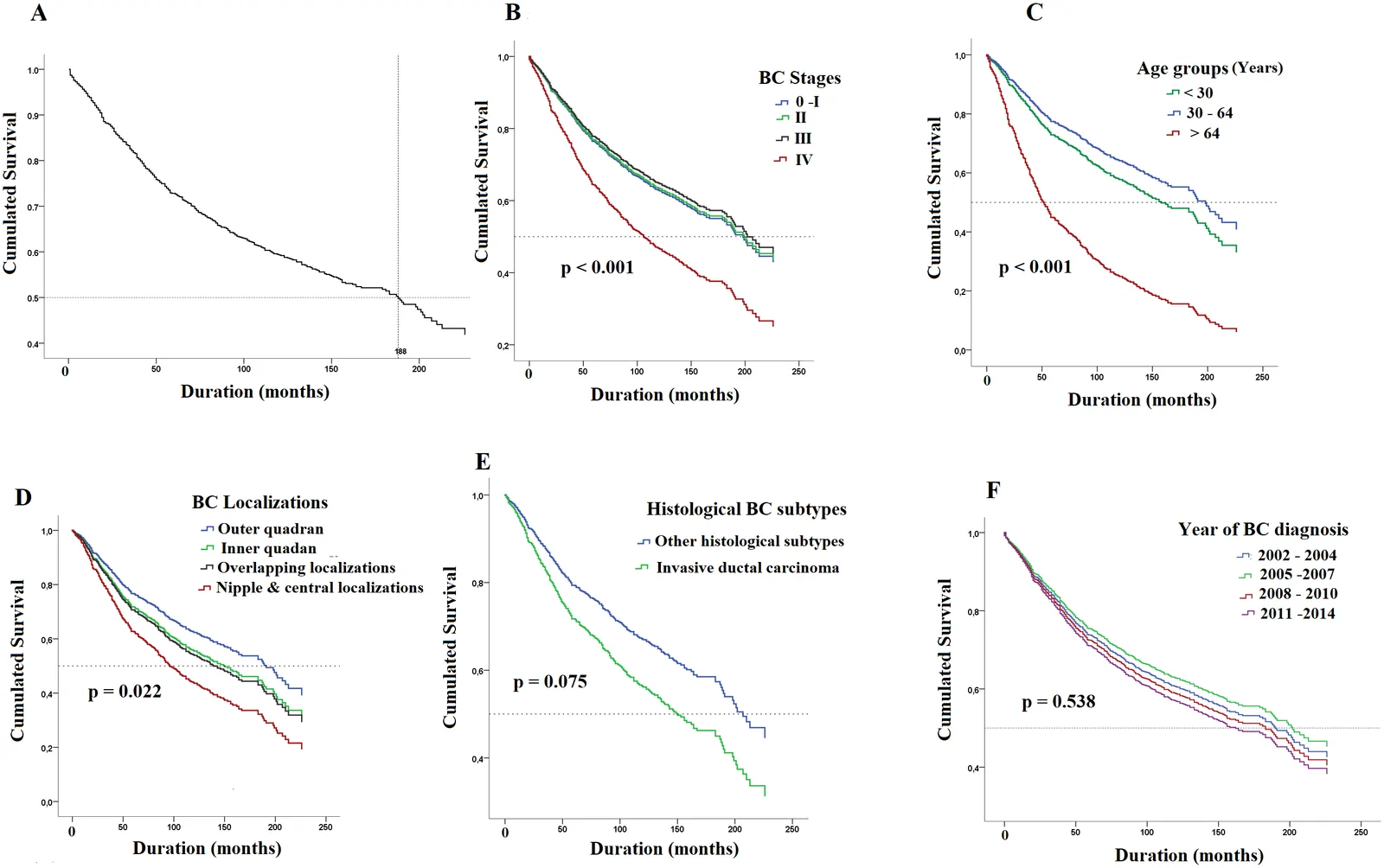
Breast cancer survival curves (Monastir, Tunisia). **A)** Overall survival of the total cohort over the follow-up period. **B)** Survival stratified by BC stage: patients with stage 4 had the poorest survival outcomes compared with other stages (p < 0.001). **C)** Survival stratified by age groups: survival significantly decreased with increasing age (p < 0.001). **D)** Survival stratified by BC topography: patients with nipple and central localizations showed lower survival rates compared with other tumor locations (log-rank test, p = 0.022). **E)** Survival stratified by BC histological subtype: no significant differences in survival were observed among histological subtypes (p = 0.075). **F)** Survival stratified by year of BC diagnosis: no significant temporal differences in survival were observed across diagnostic periods (log-rank test, p = 0.538).

According to the multivariate analysis women aged more than 64 years had a higher hazard of mortality (HR = 2.53, 95% CI: 1.17–5.48; p = 0.018). Higher mortality hazards were significantly associated with tumors in the nipple &central localizations (HR = 1.36; 95% CI: 1.06–1.75; p = 0.017) and those overlapping localizations (HR = 1.74;95% CI: 1.11–2.72; p = 0.015). Furthermore, invasive ductal carcinoma was significantly associated with a higher hazard of mortality than the reference histological subtypes, including mucinous carcinoma and Paget disease of the nipple. (HR = 1.44, 95% CI: 1.029–2.02; p = 0.035). The proportional hazards assumption evaluated graphically (log-minus-log survival curves) was found to be satisfied (S4 Appendix).

No statistically significant differences in survival were observed across cancer stages within the two age groups (<40 years and >64 years). Among the age group 40–64 years, patients with stage IV disease had significantly poorer survival (p < 0.001) (Fig 3).

**Fig 3:**
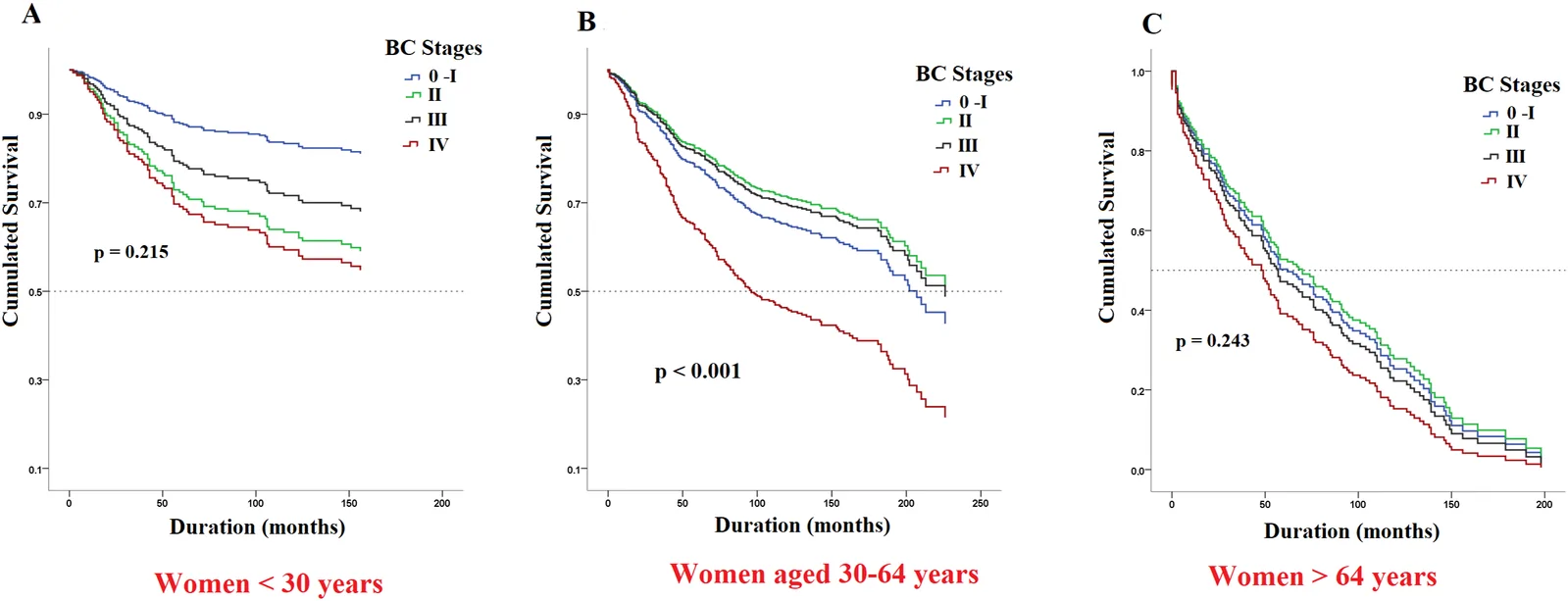
Breast cancer survival curves according to disease stage stratified by age group. (Monastir, Tunisia). **A)** Breast cancer survival curves according to disease stage among women aged <40 years; **B)** Breast cancer survival curves according to disease stage among women aged 40–64 years; **C)** Breast cancer survival curves according to disease stage among women aged >64 years.

#### 2. Three-, five- and ten-year survival rates.

Three-, five-, and ten-year survival rates were 77.0% (95% CI: 76.0–78.0), 70.0% (95% CI: 69.0–71.0), and 45.2% (95% CI: 43.2–47.2), respectively. A decline in survival was observed with increasing age. In older, the five- and ten-year survival rate were 43.5% and 4.8%. According to BC stage, ten-year survival rates were 53.6% in patients with stage 0-I and 25.9% in stage IV (Table 3).

**Table 3:** Three-, five-and ten-year survival rates of breast cancer patients by age group in Monastir, Tunisia.

	N	Three -year survival rate (95%CI)	N	Five-year survival rate (95%CI)	N	Ten-year survival rate (95%CI)
Overall	777	77.0 (76.0–78.0)	684	70.0 (69.0–71.0)	430	45.2 (43.2–47.2)
**Age groups**						
< 30	30	79.4 (72.4–86.4)	27	79.4 (72.4–86.4)	20	75.8 (68.3–83.3)
30-64	636	81.1 (80.1–82.1)	574	74.7 (73.1–76.3)	376	64.3 (62.5–66.1)
≥ 65	105	59.0 (55.0–63.0)	77	43.5 (39.5–47.5)	29	4.8 (2.8–6.8)
**BC stage**						
0-I	101	78.0 (74.0–82.0)	88	72.1 (68.1–78.1)	52	53.6 (47.6–59.6)
II	374	80.4 (78.4–82.4)	341	72.7 (70.7–74.7)	229	47.5 (44.5–50.5)
III	135	83.7 (80.7–86.7)	125	78.5 (75.5–81.5)	75	54.2 (49.8–59.2)
IV	144	61.2 (58.2–64.2)	108	52.5 (48.5–56.5)	59	25.9 (21.9–29.9)

95% CI: 95% confidence interval; N:population at risk at 3-year, 5-year and 10-year (survival analysis).

#### 3. Trends in five and ten-year survival rates.

The APCs in three- and five-year relative survival remained stable over time, whereas ten-year relative survival showed a significant upward trend, particularly among women aged 30–64 years the target population of the national BC early diagnosis program (APC: 5.374 (95%CI: 2.03; 8.82) (Table 4; Fig 4).

**Table 4:** Trends in Five-year and ten-year survival rates of breast cancer patients by age group in Monastir, Tunisia.

	Period at diagnosis	Three-year SR APC (95%CI)	p	Five-year SR APC (95%CI)	p	Period at diagnosis	Ten-year SR APC (95%CI)	p
**Overall**	2002-2014	0.579 (−0.84;2.77)	0.352	0.260 (−1.87; 2.37)	0.748	2002 − 20011	4.86 (0.002; 8.93)	0.05
**Age groups**								
30–64 years	2002-2014	0.463 (−0.88; 1.80)	0.465	0.614 (−1.11; 2.37)	0.436	2002 − 2011	5.374 (2.03; 8.82)	0.005
≥ 65 years	2002-2014	−2.42 (−6.56; 1.14)	0.165	−1.704 (−4.29; 1.94)	0.321	2002 − 2011	15.75 (−4.29; 38.80)	0.147

APC: Annual Percent Change; 95% CI: 95% confidence interval; SR: Survival Rates.

**Fig 4:**
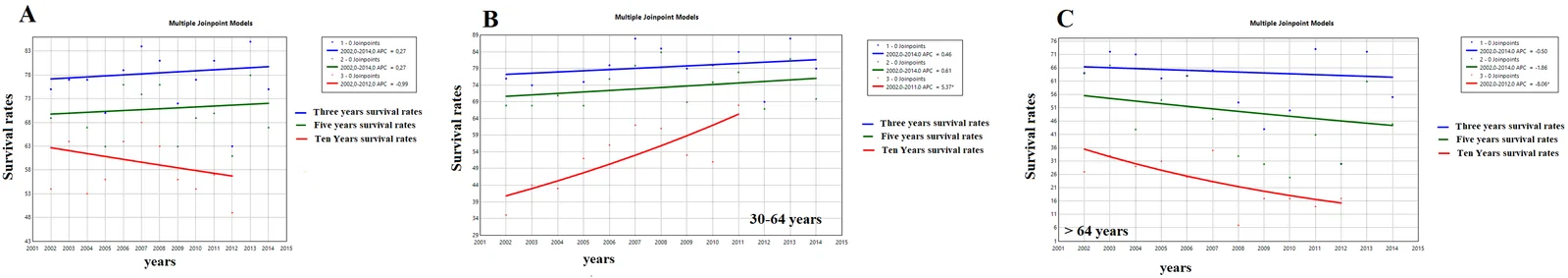
Temporal trends in three-, five-, and ten-year breast cancer survival rates in Monastir, Tunisia (2002–2022), according to age group. **(A)** Overall survival trends. **(B)** Survival trends in patients aged 40 - 65 years: showed a positive significant temporal trend in ten-year survival. **(C)** Survival trends in patients aged >65 years.

#### 4. Disability-adjusted life years (DALYs) in breast cancer.

The total BC burden was estimated at 11,800.5 DALYs, including 10,362 YLLs and 1,438.5 YLDs (Table 5). The median DALYS was 2.7 years (IQR: 1.8–23.0) (Fig 5).

**Table 5:** Disability-Adjusted Life Years (DALYs) of breast cancer according to age group in Monastir, Tunisia.

	DALY	YLL	YLD	YLL/DALY (%)
Overall (Σ)	11,800.5	10,362	1,438.5	87.8
**Age groups $**				
< 30	486.9	421	65.9	86.5
30–39	3088.9	2793	296.0	90.4
40–49	3,923.5	3423	500.5	87.2
50–59	2,635.6	2325	310.6	88.2
60–69	1,221.1	1042	179.1	85.3
≥ 70	444.4	358*	86.4	80.6

DALY: Disability-Adjusted Life Years; YLL: Years of Life Lost; YLD: Years Lived with Disability.

**Fig 5:**
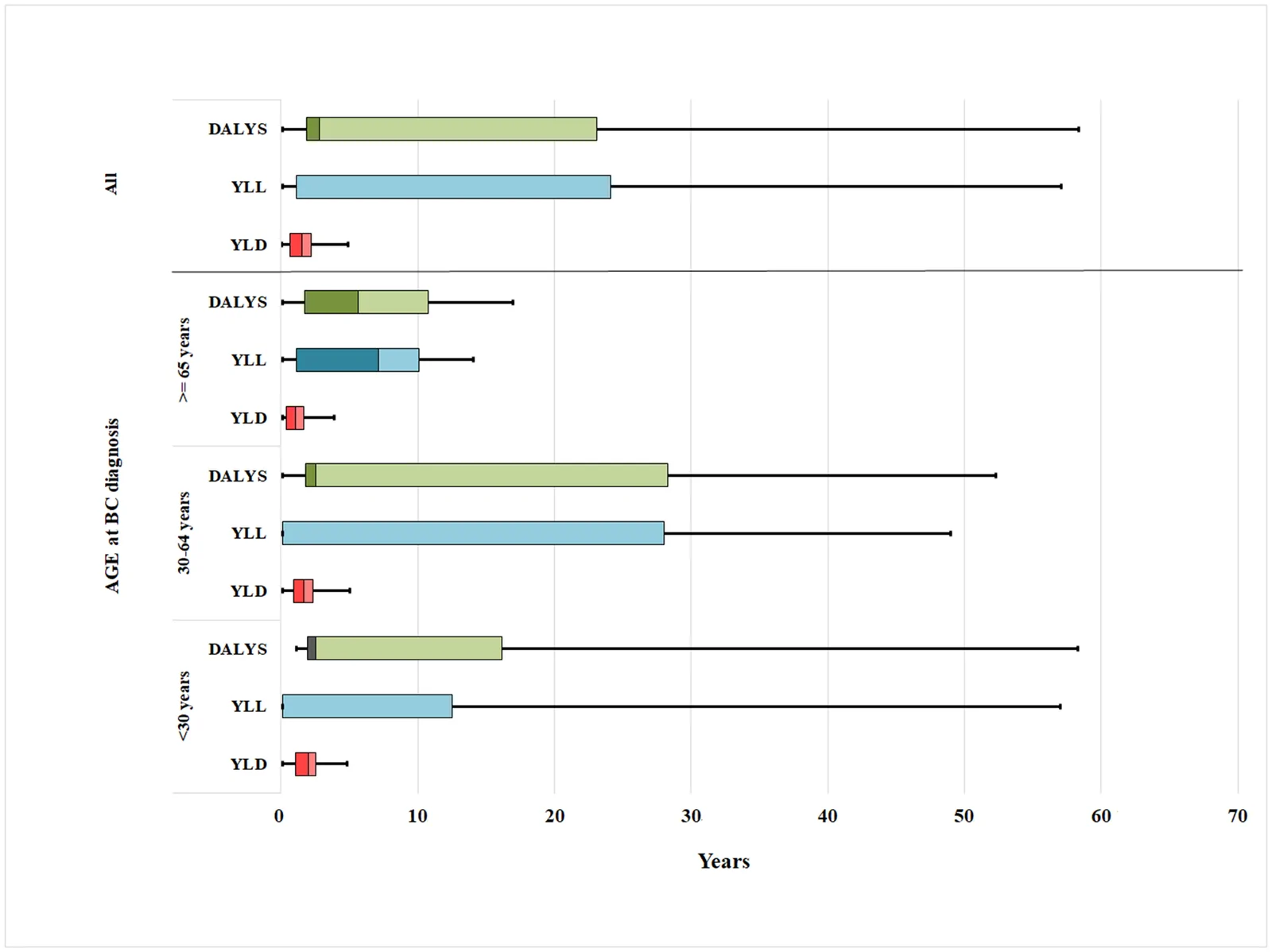
Distribution of the burden of disease among breast cancer patients. 2002–2014 (Monastir, Tunisia). Distribution of DALYsfor all women, (median of 2.68 years (IQR: 1.76–23.0), and according to age groups. Women diagnosed before the age of 30 years experienced the highest YLL and DALYs.

#### 5. Effect of Early-Stage Diagnosis (Stages 0–II) on survival and disease burden.

Compared with women diagnosed at stages III-IV, those diagnosed at stages 0-II had significantly lower 10-year mortality, with a 22% reduction in the relative risk of death (RR = 0.78; 95% CI: 0.67–0.91; p = 0.001). Diagnosing nine women at stages 0-II rather than stages III–IV would prevent approximately one additional death over the 10-year follow-up period.

This benefit was consistent for 10-year survival among women aged 30–64 years (RRR = 24.1%; NND = 9). For 5-year survival in the overall study population (RRR = 24.0%; NNT = 10). Regarding disease burden, diagnosing 61 women at stages 0-II rather than stages III-IV avoid 1 DALY. (Table 6).

**Table 6:** Relative risk, relative risk reduction, and number needed to early diagnosis for mortality according to breast cancer stage (Monastir, Tunisia).

	Death		all					
	Yes (n)	No (n)		RR	95% CI (RR)	p	RRR, % (95% CI)	NNT
**Ten-years survival rates**								
*Overall*								
Stage 0-II	216	354	570	1		0.001	22 (9.3–32.9)	9 (6–24)
Stage III-IV	171	181	352	0.780	0.671–0.907			
*At Age group 30–64 years*								
Stage 0-II	145	316	461	1				
Stage III-IV	111	157	268	0.759	0.62–0.92	0.006	24.1 (8–38)	10 (6–36)
**Five-years survival rates**								
Stage 0-II	156	414	570	1		0.005	24 (8–37)	11 (7–38)
Stage III-IV	127	225	352	0.759	0.626–0.920			
**DALYs**								
Stage 0-II	5323	593		1				
Stage III-IV	3933	361		0.982	0.970–0.995	0.005		61 (36–211)

RRs: Relative risks; RRRs: relative risk reductions; NND: Numbers Needed to Diagnosis.

### B. Breast cancer in males

Among male patients, 18 of 43 (41.9%) died during follow-up. The crude mortality rate (CMR) was 0.48 per 100,000 person-years (95% CI: 0.30–0.70). Median survival was 203 months (95% CI: 132.8–273.2). Three-, five-, and ten-year survival rates were 82.5% (95% CI: 76.5–88.5), 75.0% (95% CI: 69.2–78.8), and 67.3% (95% CI: 59.8–74.8), respectively. DALYs were 241.2, comprising 230 YLLs and 74.6 YLDs.

## Discussion

### Main results

Among 1,075 incident BC cases identified between 2002 and 2014, 21.4% were diagnosed at stage IV. By 2022, vital status information was available for 959 women. The CMR and ASMR were 11.9 (95% CI: 8.5–15.4) and 12.5 (95% CI: 8.9–16.0) per 100,000 WY, respectively. Median survival was 187 months (95% CI: 163.7–210.2). It was 226 months among women with stage 0–I BC. Three-, five-, and ten-year survival rates were 77.0%, 70.0%, and 45.2%, respectively. The APCs in ten-year survival showed a significant upward trend among the target population of the national BC screening program (women aged 30–64 years) (APC: 5.374 (95%CI: 2.03; 8.82). The total BC burden was 11,800.5 DALYs, of which 10,362 YLLs (87.8%). Median DALYs were 2.7 years (IQR: 1.8–23.0).. Diagnosis at stages 0–II was associated with a 22% reduction in ten-year mortality (RR = 0.78; 95% CI: 0.67–0.91), with an NND of 9. Diagnosing 61 women at stages 0–II rather than III–IV would avoid one DALY.

### Interpretation

Over the 20-year study period, in Monastir governorate, the mean ASMR was 12.5 (95% CI: 8.9–16.0) per 100,000 WY, lowest than the BC ASMR estimates reported for Tunisia [22,23]. This difference may be interpreted by the paucity of population-based BC mortality studies in Tunisia [24].. Patients diagnosed during the earlier years of the study were more likely to have incomplete follow-up than those diagnosed more recently. Nevertheless, survival did not differ significantly according to the year of diagnosis (log-rank p = 0.538), suggesting that the higher proportion of missing follow-up among earlier cases is unlikely to have materially influenced the overall survival estimates. Residual bias related to incomplete follow-up and unmeasured confounding factors cannot be entirely excluded.

In our cohort, the overall BC median survival was 187 months, with three-, five- and ten-year survival rates at 77.0%; 70.0% and 45.2%, respectively. Higher survival outcomes have been consistently reported in European countries, largely attributable to the implementation of widespread screening programs and early-stage diagnosis [25]. In addition, high income countries benefit from well-structured healthcare systems, broad access to specialized treatments, and comprehensive follow-up care, all of which contribute to improved long-term survival. In Asia, survival outcomes vary considerably between high-income and lower-resource settings. Developed countries such as Japan and South Korea report survival rates comparable to, or even exceeding, European averages, mainly due to national screening strategies and advances in personalized therapies [25]. In contrast, lower-resource Asian countries continue to experience poorer survival outcomes, reflecting persistent disparities in early detection, access to treatment, and socioeconomic conditions [25].

Our results were slightly higher than rates reported in other North African countries such as Algeria and Libya [26,27]. Five-year relative survival for BC among women in Algeria was 38.5% during 1990–1994. Given the broadly similar living conditions in Algeria and Tunisia, this finding may suggest an improvement in BC management between the 1990s and the 2000s in North Africa [26]. However, these survival rates remain lower than those reported in high-income countries, where five-year survival rates exceed 80%. This disparity may reflect differences in the implementation of well-established screening programs, access to state-of-the-art diagnostic and therapeutic care, and the availability of health resources. It also underscores the potential contribution of socioeconomic inequalities and limited access to cancer care, including disparities between urban and rural populations [28–34].

Although breast cancer in young women has been associated with more aggressive pathological features [35,36] and greater psychosocial difficulties [37,38], our results did not show significantly poorer survival in this age group. This finding may partly reflect more intensive treatment and closer family support among younger women, potentially contributing to better treatment adherence and continuity of care.

Women aged >64 years compared to other age groups, tumors in the nipple &central localizations and those overlapping localizations compared to other localizations, invasive ductal carcinoma compared to mucinous carcinoma and Paget disease of the nipple had factors associated with mortality. However, these findings should be interpreted with caution, as factors associated with mortality, including treatment modalities, tumor biological characteristics, and socioeconomic factors such as educational level and social status, were unavailable in the cancer registry [39,40]. The particularly poor survival observed among women aged >64 years deserves specific attention. Our stage-stratified analysis confirmed poorer survival in this age group, suggesting that the survival disadvantage of older women. Although our registry does not contain treatment data and therefore cannot establish whether older women received less intensive surgical or systemic treatment, undertreatment or differences in access to recommended therapies may contribute to these disparities. The age range covered by the organized screening strategy may also be relevant, as women older than 64 years are outside the current target population for systematic screening and may therefore be more likely to present at a later stage. These findings highlight an important public health concern regarding equity in early detection and access to appropriate BC management among older women [41]. The no significant improvement in three and five years survival might be due to the inability to implement targeted therapies (as Trastuzumab) consistently across the population during 2002–2014. The trend toward improved 10-year survival among women targeted by the national BC control program (aged 30–64 years) may reflect the program’s contribution to improved BC outcomes. This finding underscores the value of maintaining and continuously strengthening such programs through sustained investment in human and material resources, particularly in low- and middle-income settings. In contrast, a declining trend in 10-year survival was observed among women aged ≥65 years, potentially reflecting greater difficulties in accessing timely and appropriate cancer treatment in this older population [42,43]. Temporal trend analyses should be interpreted with caution, particularly in patients aged ≥ 65 years, for whom the relatively small sample size resulted in wide CI around the APC estimates. Larger studies based on a national population-based cancer registry would improve statistical precision and provide greater power to detect modest temporal changes.

In our study, the total BC burden was high, YLL accounted for 87.8% of total DALYs. These findings are consistent with international evidence showing substantial variations in the composition of DALYs according to countries’ socioeconomic development and healthcare system performance. In high-income countries, the proportion of YLD tends to be higher because of earlier diagnosis, improved access to treatment, and prolonged survival. In Norway, YLD account for approximately 38% of BC DALYs. Conversely, in low-income settings, as our results, premature mortality remains the dominant contributor to disease burden, as illustrated in Uganda, where 11% of BC DALYs are attributable to YLD [19,44]. Among women aged <50 years, the higher number of YLL may be attributable to younger age at death, which increases the difference between age at death and remaining life expectancy. Similar age-related patterns have been reported in studies from the Middle East and North Africa region [44]. These findings indicate that earlier diagnosis, the development of age-specific BC control strategies and the improved treatment access would have a substantially greater impact on reducing DALYs in Tunisia. In our study, diagnosis at an early stage (0–II) was associated with a 22% reduction in 10-year mortality compared with diagnosis at stages III-IV. This finding is consistent with evidence showing substantially better long-term survival when BC is detected at a localized rather than advanced stage [33,45]. The NND of 9 underscores the potential population-level benefit of earlier diagnosis, while the estimated avoidance of one DALY for every 61 women diagnosed at stages 0–II further supports strengthening clinical breast examination as an accessible approach to early detection [5,45,46]. Despite its limited sensitivity, CBE has demonstrated value in a low-middle-income country. Targeted awareness-raising efforts are essential to increase the use of BC screening and early detection methods [47]. Further research should evaluate complementary, non-invasive, with a better sensitivity, and acceptable early-detection strategies adapted to socioeconomic barriers. While preserving the progress made among young and middle-aged women, future policies should also prioritize improving BC care for older women. Strengthening population-based cancer registries and regularly monitoring survival trends by age group will be essential to assess the impact of these interventions and guide national cancer control policies.

### Strengths and limitations

This study has several strengths, including its population-based design, long-term follow-up, and comprehensive case ascertainment, providing robust real-world estimates of BC survival. Beyond survival outcomes, it also quantified the burden of disease using disability-adjusted life years (DALYs) and years of life lost (YLLs), generating valuable evidence to inform cancer control strategies in Tunisia. Several limitations should nevertheless be acknowledged. Of the 1,075 incident BC cases, 959 were included in the survival analysis. Sensitivity analyses showed no significant differences between women included in the analysis and those with missing vital status, suggesting that selection bias was likely limited. Nevertheless, ascertainment of vital status may have been influenced by sociocultural factors, potentially contributing to incomplete vital status ascertainment. To estimate crude and age-standardized mortality rates, cases with missing vital status were imputed according to the observed age-specific mortality distribution to preserve the population mortality structure. Although linkage of the population-based cancer registry with hospital, mortality, and administrative databases ensured high case completeness and prolonged follow-up, these sources lacked information on treatment, molecular subtype, comorbidities, recurrence, and disease progression, limiting analyses to baseline clinic-pathological characteristics. Other limitation is that the proportional hazards assumption was assessed by visual inspection of log-minus-log survival plots rather than by a formal statistical test based on Schoenfeld residuals. The estimation of YLDs also has inherent limitations. DW were assigned according to stage at diagnosis using published stage-specific estimates and assumed to remain constant throughout follow-up because information on changes in disease status was unavailable. This approach may overestimate disability among long-term survivors with early-stage disease and underestimate disability in patients experiencing progression. Furthermore, the DW used were derived from a Chinese population and may not fully represent disability patterns in Tunisia. Therefore, YLD and consequently DALY estimates should be interpreted with caution. In contrast, YLL does not depend on disability weights and therefore represents the more robust component of the DALY estimates. Finally, as the study was conducted in the governorate of Monastir, caution is warranted when generalizing the findings to other regions of Tunisia. Nevertheless, Monastir hosts one of the country’s major university medical centers and an established population-based cancer registry, making these findings an important regional benchmark and a valuable contribution to the limited evidence on long-term BC survival and burden in Tunisia.

### Recommendations

These findings support strengthening early detection across all age groups. In women aged 30–64 years, priority should be given to maintaining organized early-detection strategies, including clinical breast examination and appropriate mammographic assessment, together with equitable access to diagnostic services and timely treatment. A weekly BC prevention day led by midwives, based on pre-established lists of eligible women, could facilitate systematic CBE; outreach activities in workplaces and industrial settings could further improve access among working women. In women younger than 30 years, efforts should focus on BC awareness and identification of women at increased hereditary risk who may benefit from genetic counseling. Particular attention should be given to women aged ≥65 years, who showed less favorable long-term survival trends, by reducing barriers to diagnosis and treatment and ensuring individualized, multidisciplinary care. Across all age groups, reducing diagnostic and treatment delays and improving continuity of care should remain priorities, given the substantial mortality and DALY benefits associated with diagnosis at stages 0–II.

## Conclusion

This study provides population-based evidence on BC survival and burden in Tunisia over two decades. Older age was the main baseline clinic-pathological factor associated with mortality. Early-stage diagnosis was associated with substantially lower 10-year mortality. These findings support strengthening early detection, reducing diagnostic and treatment delays, and ensuring equitable, age-appropriate care, particularly for older women. Continued population-based cancer registration and routine survival monitoring are essential to assess the impact of cancer control strategies and guide evidence-based health policies in Tunisia.

## Supporting information

S1 Appendix Comparison of patients with missing and complete BC stage data according to age, disease stage, histological subtype, and tumor localization. (DOCX)

S2 Appendix Age-specific weights (wi) from the WHO World Standard Population 2001 used for survival standardization. (DOCX)

S3 Appendix Distribution of histological subtypes in the study population. (DOCX)

S4 Appendix FIG: The proportional hazards assumption was evaluated graphically using log-minus-log survival curves (it was found to be satisfied). (TIF)

## References

[1] Kim J, Harper A, McCormack V, Sung H, Houssami N, Morgan E, et al. Global patterns and trends in breast cancer incidence and mortality across 185 countries. Nat Med. 2025;31(4):1154–62. doi: 10.1038/s41591-025-03502-3 39994475

[2] Zemni I, Kacem M, Dhouib W, Bennasrallah C, Hadhri R, Abroug H. Breast cancer incidence and predictions (Monastir, Tunisia: 2002–2030): A registry-based study. PLoS One. 2022;17(5):e0268035. doi: 10.1371/journal.pone.0268035 PMC9135193 35617209

[3] Khiari H, Arfaoui E, Mahjoub N, Henchiri S, Sliti A, Hsairi M. Estimated Cancer Incidence in Northern Tunisia in 2023: Northern Tunisia Cancer Registry. Asian Pac J Cancer Prev. 2024;25(12):4359–69. doi: 10.31557/APJCP.2024.25.12.4359 39733429 PMC12008349

[4] Dhaouadi S, Cherif A, Hannachi H, Osman M, Hsairi M. Burden of breast and gynecological cancers in Tunisia between 1990 and 2030: Trend and projection. Tunis Med. 2021;99(4):390–8. 35244922 PMC8734482

[5] Sriha Belguith A, Elmhamdi S, Bouannene I, Ben Salem K, Soltani M. The clinical breast examination. An experience in general medicine. Tunis Med. 2013;91(2):104–11. 23526272

[6] Ayoub L. Disparity of the health sector in Tunisia in favour of the centre eastern region, the sahel and Sousse governorate. IJSAT. 2025;16(1):1–10. doi: 10.71097/ijsat.v16.i1.1346

[7] Sassi F, Rekaya MB, Belarbi A, Chilla D, Mansouri N, Achouri L, et al. Pathologically confirmed women’s breast cancer: A descriptive study of Tunisian and Algerian series. Cancer Rep (Hoboken). 2023;6 Suppl 1(Suppl 1):e1818. doi: 10.1002/cnr2.1818 37092543 PMC10440841

[8] Maatouk A, Kacem M, Bennasrallah C, Dhouib W, Bouatay M, Gara A, et al. Survival and Mortality of Cancer (SAMOC). 10.17504/protocols.io.81wgbw4pogpk/v1

[9] Ministry of Public Health (Tunisia). National health map (Carte sanitaire). 2011. Available from: https://santetunisie.rns.tn/fr/carte-sanitaire/carte-sanitaire-2011

[10] Dhouib W, Maatouk A, Bennasrallah C, Kacem M, Doghri S, Trigui A. Incidence and survival of lung cancer: A population-based cohort study (Monastir, Tunisia: 2002–2022). PLoS One. 2025;20(12):e0338140. 10.1371/journal.pone.0338140 PMC12680147 41348719

[11] Amin MB, Edge SB, Greene FL, Byrd DR, Brookland RK, Washington MK, et al., AJCC Cancer Staging Manual. 8th ed. New York: Springer; 2017.

[12] Altman DG, Machin D, Bryant TN, Gardner MJ. Statistics with confidence: confidence intervals and statistical guidelines. 2nd ed. London: BMJ Books; 2000.

[13] Surveillance, Epidemiology, and End Results (SEER) Program. World (WHO 2000–2025) standard populations. Available from: https://seer.cancer.gov/stdpopulations/world.who.html

[14] National Cancer Institute. NCI Dictionary of Cancer Terms. 2011. Available from: https://www.cancer.gov/publications/dictionaries/cancer-terms

[15] National Cancer Institute. Definition of five-year survival rate. 2011. Available from: https://www.cancer.gov/publications/dictionaries/cancer-terms/def/five-year-survival-rate

[16] Institut National de la Statistique (INS). Monastir à travers le recensement général de la population et de l’habitat 2014. Tunis: Institut National de la Statistique; 2016.

[17] Yan X-X, Zhu J, Li Y-J, Cao M-D, Wang X, Wang H, et al. Estimating disability-adjusted life years for breast cancer and the impact of screening in female populations in China, 2015-2030: An exploratory prevalence-based analysis applying local weights. Popul Health Metr. 2022;20(1):19. doi: 10.1186/s12963-022-00296-1 36207752 PMC9547451

[18] Wu D, Kim S. Inference of onset age of preclinical state and sojourn time for breast cancer. Med Res Arch. 2022;10(2):2665. doi: 10.18103/mra.v10i2.2665 35419489 PMC9004723

[19] Xu S, Liu Y, Zhang T, Zheng J, Lin W, Cai J, et al. The Global, Regional, and National Burden and Trends of Breast Cancer From 1990 to 2019: Results From the Global Burden of Disease Study 2019. Front Oncol. 2021;11:689562. doi: 10.3389/fonc.2021.689562 34094989 PMC8176863

[20] Global Burden of Disease 2019 Cancer Collaboration. Cancer incidence, mortality, years of life lost, years lived with disability, and disability-adjusted life years for 29 cancer groups, 2010–2019. JAMA Oncol. 2022;8(3):420–44. 10.1001/jamaoncol.2021.6987 PMC8719276 34967848

[21] Kim HJ, Fay MP, Feuer EJ, Midthune DN. Permutation tests for joinpoint regression with applications to cancer rates. Stat Med. 2000;19(3):335–51. doi: 10.1002/(sici)1097-0258(20000215)19:3<335::aid-sim336>3.0.co;2-z 10649300

[22] Sharma R. Global, regional, national burden of breast cancer in 185 countries: evidence from GLOBOCAN 2018. Breast Cancer Res Treat. 2021;187(2):557–67. doi: 10.1007/s10549-020-06083-6 33515396

[23] Sharma R, Aashima, Nanda M, Fronterre C, Sewagudde P, Ssentongo AE, et al. Mapping cancer in Africa: A comprehensive and comparable characterization of 34 cancer types using estimates from GLOBOCAN 2020. Front Public Health. 2022;10:839835. doi: 10.3389/fpubh.2022.839835 35548083 PMC9082420

[24] Badu-Peprah A, Kontor EK, Benjamin AG, Kumah J, Essuman AA, et al. Five-year survival outcomes for breast cancer patients across Africa: A systematic review and meta-analysis. eLife. 2025;14:e105488.

[25] OECD, European Commission. EU country cancer profiles: Synthesis report 2025. Paris: OECD Publishing; 2025. Available from: https://www.oecd.org/en/publications/eu-country-cancer-profiles-synthesis-report-2025_20ef03e1-en.html

[26] Hamdi-Cherif M, Benabdallah A, Sadi B, Mahnane A, Zaidi Z, Hamdi B. Cancer estimation of incidence and survival in Algeria 2014. J Cancer Res Ther. 2015;3(9):100–4.

[27] El Mistiri M, Salati M, Marcheselli L, Attia A, Habil S, Alhomri F, et al. Cancer incidence, mortality, and survival in Eastern Libya: updated report from the Benghazi Cancer Registry. Ann Epidemiol. 2015;25(8):564–8. doi: 10.1016/j.annepidem.2015.03.012 25911981

[28] American Cancer Society. Breast cancer facts & figures 2024–2025. Atlanta: ACS; 2024.

[29] Lee J, Park J, Kim N, Nari F, Bae S, Lee HJ, et al. Socioeconomic Disparities in six common cancer survival rates in South Korea: Population-wide retrospective cohort study. JMIR Public Health Surveill. 2024;10:e55011. doi: 10.2196/55011 39041282 PMC11284611

[30] Fu M, Peng Z, Wu M, Lv D, Li Y, Lyu S. Current and future burden of breast cancer in Asia: A GLOBOCAN data analysis for 2022 and 2050. Breast. 2025;79:103835. doi: 10.1016/j.breast.2024.103835 39549557 PMC11609486

[31] Mubarik S, Sharma R, Hussain SR, Iqbal M, Nawsherwan, Liu X. Breast cancer mortality trends and predictions to 2030 in East and South Asia. Front Nutr. 2022;9:847920. 10.3389/fnut.2022.847920 PMC8964109 35360680

[32] Quang DT, Luong TT, Nguyen DK, Vu VTQ, Nguyen THH, Phan NQ. Breast cancer survival among Southeast Asian women: A systematic review and meta-analysis. Curr Probl Cancer. 2024;48:101062. 10.1016/j.currproblcancer.2024.101062 38309146

[33] Corbex M, Bouzbid S, Boffetta P. Features of breast cancer in developing countries, examples from North-Africa. Eur J Cancer. 2014;50(10):1808–18. doi: 10.1016/j.ejca.2014.03.016 24767469

[34] Anders CK, Johnson R, Litton J, Phillips M, Bleyer A. Breast cancer before age 40 years. Semin Oncol. 2009;36(3):237–49. doi: 10.1053/j.seminoncol.2009.03.001 19460581 PMC2894028

[35] Cancello G, Maisonneuve P, Rotmensz N, Viale G, Mastropasqua MG, Pruneri G. Prognosis and adjuvant treatment effects in very young women with operable breast cancer. Ann Oncol. 2010;21(10):1974–81. 10.1093/annonc/mdq072 20332136

[36] Ahmad S, Fergus K, McCarthy M. Psychosocial issues experienced by young women with breast cancer. Curr Opin Support Palliat Care. 2015;9(3):271–8. doi: 10.1097/spc.0000000000000162 26147915

[37] Hasan S, Chew KS. Unmet psychosocial needs and quality of life of young women with breast cancer: A scoping systematic review. Asian Pac J Cancer Prev. 2024;25(11):3781–8. doi: 10.31557/APJCP.2024.25.11.3781 39611900 PMC11996109

[38] Costa L, Kumar R, Villarreal-Garza C, Sinha S, Saini S, Semwal J. Diagnostic delays in breast cancer among young women. Breast. 2024;73:103623. 10.1016/j.breast.2023.103623 PMC10826418 38219460

[39] Belaid I, Fatma LB, Ezzairi F, Hochlaf M, Chabchoub I, Gharbi O, et al. Trends and current challenges of breast cancer in Tunisia: A retrospective study of 1262 cases with survival analysis. Breast J. 2018;24(5):846–8. doi: 10.1111/tbj.13062 29781116

[40] Ben Fatma L, Belaid I, Said N, Gahbiche S, Hochlef M, Chabchoub I. Reasons for late diagnosis of breast cancer in Tunisia. Tunis Med. 2018;96(10–11):665–71. 30746659

[41] Kim JH, Kim YY, Chung JH, Kim WY, Lee JB, Woo SU. Breast cancer in older patients aged 70 years and above: Treatment adherence and oncologic outcomes. J Breast Cancer. 2025;28(2):99. doi: 10.4048/jbc.2024.0190 PMC12046351 40133983

[42] Meriam K, Manel L, Jihene S, Hédi K, Ali M, Thouraya A. Delays in the surgical pathway in breast cancer patients in Tunisia. Indian J Surg Oncol. 2022;13(2):385–393. 10.1007/s13193-021-01484-5 PMC9240175 35782809

[43] Soerjomataram I, Lortet-Tieulent J, Ferlay J, Forman D, Mathers C, Parkin DM, et al. Estimating and validating disability-adjusted life years at the global level: A methodological framework for cancer. BMC Med Res Methodol. 2012;12:125. doi: 10.1186/1471-2288-12-125 22901001 PMC3490831

[44] Safiri S, Noori M, Nejadghaderi SA, Sullman MJM, Bragazzi NL, Almasi-Hashiani A, et al. Burden of female breast cancer in the Middle East and North Africa region, 1990–2019. Arch Public Health. 2022;80(1):168. 10.1186/s13690-022-00918-y PMC9272597 35818086

[45] Patient navigation for early detection, diagnosis and treatment of breast cancer: technical brief. [cited 2026 Août 10]. Available from: https://www.who.int/publications/i/item/9789240100954

[46] Mittra I, Mishra GA, Dikshit RP, Gupta S, Kulkarni VY, Shaikh HKA, et al. Effect of screening by clinical breast examination on breast cancer incidence and mortality after 20 years: prospective, cluster randomised controlled trial in Mumbai. BMJ. 2021;372:n256. doi: 10.1136/bmj.n256 33627312 PMC7903383

[47] Bennasrallah C, Youssef YB, Kacem M, Maatouk A, Dhouib W, Fredj MB, et al. Factors associated with breast self-examination and mammography screening among women attending primary healthcare centers in central Tunisia. BMC Womens Health. 2026;26(1):451. doi: 10.1186/s12905-026-04623-8 42363129 PMC13563729

