# Supplementary material for: Survival and disease burden of breast cancer (Monastir, Tunisia): A population-based cohort study (2002–2022)
Source: PLoS One. 2026 Oct 14;21(10):e0360398. doi: 10.1371/journal.pone.0360398 (PMC13667823; doi:10.1371/journal.pone.0360398)
Supplement: Supplementary file 1 [file pone.0360398.s001.docx]

**Appendix Table S1**

**Comparison of patients with missing and complete stage data according to age, histological subtype, and tumor localization**

| **Age groups** | **Missing data** | **Valid data** | **p** |
| --- | --- | --- | --- |
| < 30 | 2 (5.6) | 34 (94.8) | 0.334 |
|  | 9 (4.6) | 188 (95.4) |  |
| 40-49 | 7 (2.3) | 303 (97.7) |  |
| 50 -59 | 12 (4.9) | 234 (95.1) |  |
| 69 | 9 (6.2) | 137 (93.8) |  |
| ≥ 70 | 3 (2.8) | 105 (97.2) |  |
| **BC localizations** |  |  |  |
| Nipple &central localizations | 1 (2.6) | 38 (97.4) | 0.630 |
| Upper-inner quadrant | 6 (6.5) | 86 (93.5) |  |
| Lower-inner quadrant | 2 (4.9) | 39 (95.1) |  |
| Upper-outer quadrant | 11 (4.3) | 247 (95.7) |  |
| Lower-outer quadrant | 1 (1.6) | 60 (98.4) |  |
| **BC histological tumor types** |  |  |  |
| Invasive ductal carcinoma (IDC) | 31 (4.4) | 671 (95.6) | 0.118 |
| Other subtypes | 10 (7.6) | 121 (92.4) |  |
| **Year of diagnosis** |  |  |  |
| 2002-2004 | 9 (5.7) | 148 (94.3) | 0.677 |
| 2005-2007 | 13 (5.6) | 221 (94.4) |  |
| 2008-2010 | 22 (7.6) | 269 (92.4) |  |
| 2011-2014 | 30 (7.6) | 363 (92.4) |  |
