# Supplementary material for: Survival and disease burden of breast cancer (Monastir, Tunisia): A population-based cohort study (2002–2022)
Source: PLoS One. 2026 Oct 14;21(10):e0360398. doi: 10.1371/journal.pone.0360398 (PMC13667823; doi:10.1371/journal.pone.0360398)
Supplement: Supplementary file 2 [file pone.0360398.s002.docx]

**Appendix 1. Age-specific weights (w_i_) from the WHO World Standard Population 2001 used for survival standardization**

**Age group (years)	Corresponding weight (Wi)***

**<30	0.304**

**30–39	0.155**

**40–49	0.134**

**50–59	0.110**

**60–69	0.086**

**≥70	0.211**

***** *Weights are aggregated from 5-year age groups of the WHO 2001 World Standard Population to match the study’s age categories. The sum of weights equals 1***.**
