# Supplementary material for: Survival and disease burden of breast cancer (Monastir, Tunisia): A population-based cohort study (2002–2022)
Source: PLoS One. 2026 Oct 14;21(10):e0360398. doi: 10.1371/journal.pone.0360398 (PMC13667823; doi:10.1371/journal.pone.0360398)
Supplement: Supplementary file 3 [file pone.0360398.s003.docx]

# Appendix Table S3

**Distribution of histological subtypes in the study population**

| **Histological type** | **ICD-O-3 codes** | **N (%)** |
| --- | --- | --- |
| Invasive ductal carcinoma (IDC) | 8500, 8501, 8503, 8523 | 717 (84.4) |
| Invasive lobular carcinoma (ILC) | 8520, 8522 | 52 (6.1) |
| Mucinous carcinoma | 8480 | 18 (2.1) |
| Paget disease of the nipple with invasive carcinoma | 8541 | 18 (2.1) |
| Carcinoma, NOS | 8010 | 3 |
| Giant cell carcinoma | 8032 | 1 |
| Papillary carcinoma, NOS | 8050 | 1 |
| Lymphoepithelial carcinoma | 8082 | 1 |
| Scirrhous adenocarcinoma | 8131 | 2 |
| Adenocarcinoma, NOS | 8140 | 5 |
| Tubular adenocarcinoma | 8211 | 2 |
| Neuroendocrine carcinoma | 8246 | 1 |
| Apocrine adenocarcinoma | 8401 | 3 |
| Medullary carcinoma | 8510 | 7 |
| Medullary carcinoma with lymphoid stroma | 8513 | 1 |
| Metaplastic carcinoma | 8573 | 3 |
| Metaplastic carcinoma | 8575 | 2 |
| Sarcoma, NOS | 8800 | 2 |
| Spindle cell sarcoma | 8801 | 1 |
| Leiomyosarcoma | 8890 | 1 |
| Carcinosarcoma | 8980 | 1 |
| Phyllodes tumor, malignant | 9020 | 5 |
| Diffuse large B-cell lymphoma | 9680 | 3 |
| Total |  | 850 |
